# Induction of salt tolerance in *Brassica rapa* by nitric oxide treatment

**DOI:** 10.3389/fpls.2022.995837

**Published:** 2022-11-16

**Authors:** Atiyyah Bano, Zahra Noreen, Fariha Tabassum, Fizza Zafar, Madiha Rashid, Muhammad Aslam, Anis Ali Shah, Adnan Noor Shah, Mariusz Jaremko, Noura Alasmael, Nader R. Abdelsalam, Mohamed E. Hasan

**Affiliations:** ^1^ Department of Botany, Division of Science and Technology, University of Education, Lahore, Pakistan; ^2^ Department of Botany, Lahore College for Women University, Lahore, Pakistan; ^3^ Department of Chemistry, Division of Science and Technology, University of Education, Lahore, Pakistan; ^4^ Department of Agricultural Engineering, Khwaja Fareed University of Engineering and Information Technology, Rahim Yar Khan, Pakistan; ^5^ Smart-Health Initiative and Red Sea Research Center, Division of Biological and Environmental Sciences and Engineering, King Abdullah University of Science and Technology, Thuwal, Saudi Arabia; ^6^ Smart Hybrid Materials Laboratory, Physical Science and Engineering Division, King Abdullah University of Science and Technology, Thuwal, Saudi Arabia; ^7^ Agricultural Botany Department, Faculty of Agriculture (Saba Basha), Alexandria University, Alexandria, Egypt; ^8^ Bioinformatics Department, Genetic Engineering and Biotechnology Research Institute, University of Sadat City, Sadat City, Egypt

**Keywords:** antioxidant, *Brassica rapa*, ecotypes, nitric oxide, salt stress, turnip, stress, phenolic

## Abstract

Salinity is one of the major plant abiotic stresses increasing over time worldwide. The most important biological role of nitric oxide (NO) in plants is related to their development and growth under abiotic conditions. The present experiment was conducted to study the effect of salt stress (0 and 100 mM) and NO (0 and 80 μM) on two different ecotypes of *Brassica rapa* (L.): PTWG-HL and PTWG-PK. The different growth attributes, biochemical and physiological parameters, and the mineral contents were examined. The results indicated increased hydrogen peroxide (H_2_O_2_), relative membrane permeability, malondialdehyde (MDA), and Na^+^ content and decreased plant biomass in both ecotypes (PTWG-PK and PTWG-HL) under salt stress. In contrast, NO treatment resulted in increased plant biomass, chlorophyll content, and total soluble proteins and decreased H_2_O_2_, relative membrane permeability, MDA, total phenolic content, catalase (CAT), peroxidase (POD), ascorbate peroxidase (APX), and Na^+^. The combined effect of salt stress and NO application increased the chlorophyll a content, total phenolic content, and total soluble proteins, but decreased H_2_O_2_, relative membrane permeability, MDA, and Na^+^. The response of carotenoids, anthocyanins, and K^+^, Ca^2+^, and Cl^−^ ions varied in both ecotypes under all treatment conditions. The PTWG-PK ecotype showed maximum overall growth response with the application of NO. Henceforth, it is proposed that the molecular mechanisms associated with NO-induced stress tolerance in plants may be exploited to attain sustainability in agriculture under changing climate scenarios.

## Introduction

According to an estimate of the economic department of the United Nations, the world has to feed 8.5 billion people by 2030 (FAO, 2014). Pakistan is ranked 17th in the production of turnip (Ali et al., 2018). The rate of turnip production contributed by Pakistan in the world market is 1.2% per year; however, turnip is among the vegetables with a low yield compared to that of other countries (Akhtar et al., 2013). One major reason for the lower yield is the presence of abiotic factors, such as salt stress (Khan et al., 2012). Many greenhouse studies have been carried out on vegetable species that have shown a decrease in plant growth under salt stress (Sogoni et al., 2021). Salt stress ultimately reduced the fresh and dry biomass collection (Colla et al., 2013a), induced deterioration of the membrane integrity (Colla et al., 2013b), reduced the synthesis of chlorophyll contents (Rouphael et al., 2017a), and restricted photosynthetic CO_2_ fixation (Rouphael et al., 2017b). The vegetable industry in Pakistan is trying to overcome the problem of salt stress *via* the selection of vegetable ecotypes and then growing the vegetables in a greenhouse (Shaukat, 2014).

The deleterious effects of salt stress on plants include limited nutrient uptake, ionic imbalance, and reduction in growth (Hussain et al., 2018). Salt toxicity reduces the growth and physiological activities of many *Brassica* species (Shahzad et al., 2022). Plants have developed defense mechanisms for tolerance to abiotic stresses (Waqas et al., 2019). Sodium nitroprusside (SNP) is primarily used as a nitric oxide (NO) donor in plant experiments and requires an electron reduction or irradiation with light in order to release NO in plants (Feelisch, 1998). NO is one of the crucial biomolecules involved in stress tolerance in plants. This biomolecule also plays a vital role in many processes of plant development (Mostofa et al., 2015). NO is involved in the positive regulation of plants subjected to drought, heavy metal, and salinity stress. This redox molecule also protects plants from oxidative damage by activating their antioxidative defense system (Nabi et al., 2019). Furthermore, increased concentration of NO in plants under salinity stress helps regulate various physiological processes. These physiological processes include seed germination, photosynthesis, nutrient uptake, osmotic balance, oxidative stress, respiration, and gene expression (Shang et al., 2022).

Salt stress adversely affects the growth and physiochemical characteristics of *Brassica rapa* (Mittal et al., 2018). In the above context, the current research was carried out to explore the potential of NO in reducing salinity stress in *B. rapa*. Moreover, the effects of NO on the growth and physiochemical attributes of *B. rapa* exposed to salt stress were studied.

## Materials and methods

Experiments were conducted on two ecotypes of the *B. rapa* cultivar Purple Top White Globe to determine the morphophysiological roles of NO addition (0 and 80 μM) in ameliorating salt stress (0 and 100 mM). Seeds of the first ecotype (PTWG-PK) were taken from Ayub Agricultural Research Institute of the University of Agriculture, Faisalabad, while the second ecotype (PTWG-HL) was imported from Holland under the brand name Hollander. Fifteen seeds of each ecotype were sown in pots containing 10 kg well washed river sand and 2 L/pot of modified Hoagland’s solution.

### Fresh and dry biomass

After 4 weeks of treatment, two plants from each pot were taken carefully and their fresh biomass examined. After taking the fresh weight, the plant samples were oven dried at 70°C for 1 week followed by weighing of the dry biomass.

### Determination of chlorophyll and carotenoid contents

Determination of the chlorophyll and carotenoid contents was performed following Arnon (1949) method. Freshly grown shoots, weighing 5 g, were ground in 10 ml acetone (80%). The filtrate was collected and the absorbance of the filtrate determined at 480, 645, and 663 nm using a spectrophotometer (model T60UV; PG Instruments Limited, Lutterworth, UK).

### Estimation of anthocyanin content

Fresh shoots weighing 0.1 g were crushed on an ice bath in 5 ml phosphate buffer. The samples were then centrifuged at 4,000 rpm for 10 min at room temperature, the supernatants were collected, and the absorbance measured at 600 nm.

### Determination of the relative membrane permeability

Membrane permeability was determined using the method of Yang et al. (1996). Fresh shoots weighing 5 g were cut and immersed in test tubes containing 10 ml distilled water at room temperature. After 24 h, the test tubes were vortexed and the EC_0_ readings were taken. After placing all the test tubes for another 24 h, at 4°C temperature, a second reading was taken for EC_1_ measurement. After another 24 h, all test tubes were put in an autoclave for 60 min at 121°C and final readings were taken for EC_2_. The following formula was used to determine the relative membrane permeability (RMP):


$${\text{RMP(\%)=(EC}}_{1}-{\text{EC}}_{0}/{\text{EC}}_{2}-{\text{EC}}_{0})\times 100$$

Where, EC is the electrical conductivity at various time intervals.

### Determination of hydrogen peroxide

We followed the method of Velikova et al. (2000) for the determination of H_2_O_2_. Freshly cut shoots of 5 g weight were ground in 5 ml of 0.1% trichloroacetic acid on an ice bath. After grinding the samples, the mixture was transferred into Eppendorf tubes and centrifuged (model Z326K; Hermle AG, Gosheim, Germany) at 4,000 rpm for 10 min at room temperature. The supernatants were collected and stored as plant extracts. A mixture of 0.1 ml was placed in test tubes along with 0.1 ml phosphate buffer and 2 ml of 1 M potassium iodide (KI) solution and left for half an hour at room temperature. Absorbance was then measured at 390 nm.

### Quantification of malondialdehyde content

The malondialdehyde (MDA) content was determined using the method of Health and Parker (1968). Fresh shoots of 5 g were crushed in 5 ml trichloroacetic acid (5%), the crushed leaves centrifuged at 4,000 rpm for 10 min at 4°C, and then 0.5 g tributyric acid (TBA) was dissolved in 100 ml of distilled water. Equal amounts of TBA and plant extracts were collected into test tubes and kept for 30 min at room temperature. The test tubes were then placed in a water bath at 100°C for half an hour. After heating, the test tubes were cooled down for 10 min and the absorbance measured at 532 and 600 nm. The concentration of TBARS (thiobarbituric acid reactive substances) was determined using an absorption coefficient of 155 mmol^−1^ cm^−1^. For the calculation of MDA equivalents, the following formula was used:


$${\text{MDA equivalents (nmol)=(Abs}}_{523nm}-{\text{Abs}}_{600nm})/1.56\times 105$$

where Abs_523 nm_ and Abs_600 nm_ are the absorbance values at 523 and 600 nm, respectively.

### Estimation of total phenolic content

The total phenolic content was determined using the method of Julkunen-Tiitto (1985). Of the fresh shoot samples, 0.5 g was crushed and thoroughly mixed with 80% acetone solution. The mixture was then centrifuged at 10,000 rpm for 10 min at 4°C. A dilution of 0.1 ml of the supernatants was prepared in a test tube by adding 2 ml water and 1 ml Folin–Ciocalteau phenol reagent. The test tube was then shaken vigorously, followed by addition of 5 ml of 20% sodium carbonate and making the volume up to 10 ml with distilled water. After vigorous shaking, the absorbance was then measured at 750 nm. The total phenolic content was determined as milligrams per gram of fresh leaf.

### Determination of total soluble protein

Fresh shoots of 0.5 g were crushed in 10 ml of cold phosphate buffer (50 mM, pH 7.8) on an ice bath and the mixture centrifuged at 6,000 rpm for 20 min at 4°C. The supernatants obtained were utilized for further analysis. The concentration of protein in the leaf extracts was measured following the method of Bradford (1976). About 2 ml of Bradford reagent and 0.1 ml of the leaf extracts were taken into test tubes and the mixture kept at room temperature for 5 min. The absorbance of each sample was measured at 595 nm.

### Determination of antioxidant enzyme activities

Fresh shoots of 0.5 g were crushed on an ice bath in 5 ml of cold phosphate buffer (50 mM, pH 7.8). The mixture was then centrifuged at 15,000 rpm for 20 min at 4°C. The supernatants collected were used for the determination of the activity of antioxidant enzymes.

#### Catalase and peroxidase activity

The method of Chance and Maehly (1955) was followed for the determination of the catalase (CAT) and peroxidase (POD) activity in turnip. A mixture of 3 ml of phosphate buffer (50 mM, pH 7.0) and hydrogen peroxide (5.9 mM) was prepared in a test tube for the determination of CAT activity. The reaction was initiated in a cuvette by adding 0.1 ml enzyme extract. Frequent fluctuations in the absorbance readings were noted after each 20-s time interval at 240 nm. One unit of CAT activity was equal to an absorbance change of 0.01 U/min. For the determination of POD activity, a reaction solution (3 ml) was prepared by mixing phosphate buffer (50 mM, pH 5.0), guaiacol (20 mM), and hydrogen peroxide (40 mM) in test tubes. The reaction was started with the addition of 0.1 ml enzyme extract in a cuvette. Fluctuations in absorbance were noted after 20 s at the 470-nm wavelength. One unit of POD activity was defined as a change in absorbance of 0.01 U/min. Enzyme activity was determined on the basis of soluble proteins.

#### Ascorbate peroxidase activity

The enzymatic activity of ascorbate peroxidase (APX) was calculated following the method of Nakano and Asada (1981). A 3-ml reaction solution was prepared by mixing hydrogen peroxidase (1.0 mM), ascorbate (0.25 mM), EDTA (0.1 mM), and sodium phosphate (25 mM, pH 7.0) in test tubes. The enzyme activity was started after the addition of 0.1 ml enzyme extract in the reaction solution. The hydrogen peroxide-dependent oxidation of ascorbate was calculated by observing the decrease in the absorbance level at 290 nm (*E* = 2.8 mM^−1^ cm^−1^).

### Estimation of mineral elements

We used the Chapman and Pratt (1978) method for the digestion and the estimation of minerals. Na^+^, K^+^, and Ca^+2^ cations in the shoot digests were calculated using a flame photometer, model 360 (Sherwood Scientific). To determine the chloride content in a solution, titration was performed. Silver nitrate was used as a titrant for the calculation of unknown chloride ions. Silver and chloride ions were reacted in a 1:1 molar ratio.

### Statistical analysis

Analysis of variance (ANOVA) for all parameters was conducted with the commonly used statistical program CoStat (CoHort Software; Version 6.303). Microsoft Excel was employed for the graphical representation of data.

## Results and discussion

**Figure 1:**
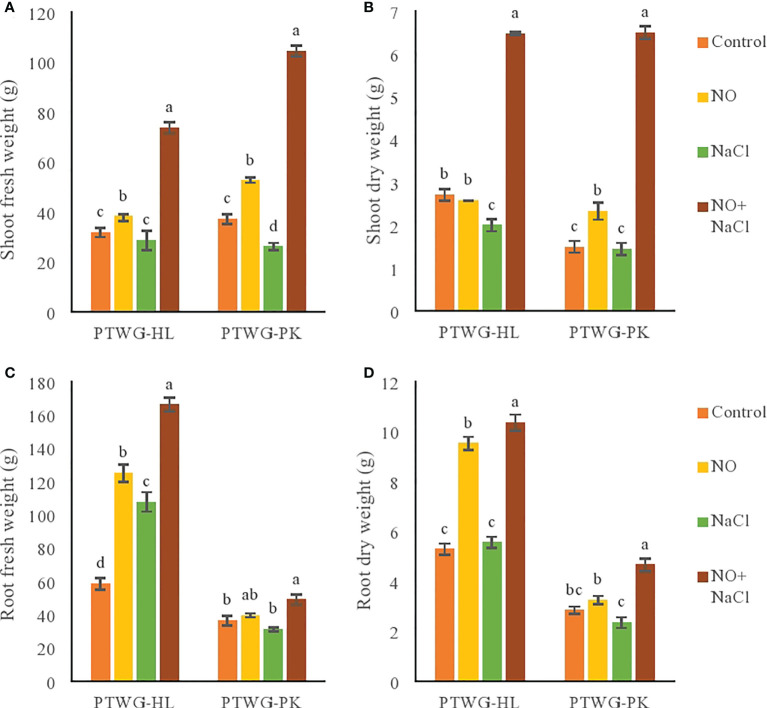
Shows fresh and dry weight of root as well as shoot of forty-two days old ecotypes of turnip (*Brassica rapa* L.) under sodium chloride and nitric oxide stress. **(A)** Shoot fresh weight, **(B)** Shoot dry weight, **(C)** Root fresh weight, **(D)** Root dry weight. Means with the same letters on each bar do not differ significantly at the 5% level.

The experiments were conducted to determine the effect of salt and NO on the morphobiochemical processes of five ecotypes of *B. rapa* L. ANOVA of the turnip ecotypes PTWG-HL and PTWG-PK exhibited that salt stress decreased the shoot and root biomass (**Table 1**). Similar findings have been previously reported in *Saccharum* sp. (Ejaz et al., 2012) and wheat (Khan and Ashraf, 2008). However, the root dry biomass of ecotype PTWG-HL was increased. A similar result was also found in the canola cultivar Liraspa (Tunçtürk et al., 2011b). The application of NO significantly increased the shoot and root biomass. Similar results were also found in *Capsicum annuum* L. (Kaya et al., 2019), mungbean (Salahuddin et al., 2017), rice (Habib and Ashraf, 2014), and wheat (Asthir et al., 2013). Salt stress and treatment with NO significantly increased the shoot and root biomass of ecotype PTWG-HL (**Figure 1**). Identical results have also been found in *Zea mays* L. (Kaya et al., 2019), tomato (Manai et al., 2014), and in cucumber (Fan et al., 2013). On the other hand, the shoot and root biomass of ecotype PTWG-PK was reduced (*p* ≤ 0.001). Recently, a study by Zhang et al. (2021) has also confirmed that NO is associated with salinity tolerance in *B. napus*.

**Table 1:** Mean square values from ANOVA of the data on the different physiological and biochemical parameters of the two ecotypes of turnip (*Brassica rapa* L.) grown for 42 days under salt stress and nitric oxide effect.

Source of variation	*df*	Shoot fresh weight = g	*p*-value	Shoot dry weight = g	*p*-value	Root fresh weight = g	*p*-value	Root dry weight = g	*p*-value	Chlorophyll a = mg/g	*p*-value
Ecotype	1	1,154.64	0	1.97	0	45,331.30	0	155.23	0	0.0008	0
Salt stress	1	2,676.19	0	26.72	0	4,456.38	0	2.05	0.0047	0.0005	0
Nitric oxide	1	10,550.90	0	52.38	0	10,633.50	0	69.03	0	0.0001	0.002
Ecotype*salt	1	36.81	0.145	0.42	0.0218	3,700.36	0	0.02	0.7842	0.0000	0.9532
Ecotype*nitric oxide	1	889.79	0	1.20	0.0004	5,379.17	0	19.94	0	0.0000	0.9764
Salt*nitric oxide	1	5,146.05	0	39.03	0	23.24	0.5262	3.08	0.0008	0.0002	0.0007
Ecotype*salt*nitric oxide	1	289.20	0.0003	0.08	0.3063	257.02	0.0428	0.92	0.0479	0.0000	0.6498
Error	24	4.03	0	0.26		7.50		0.46		0.0034	
		Chlorophyll b	*p*-value	Carotenoids	*p*-value	RMP	*p*-value	Anthocyanin	*p*-value	H_2_O_2_	*p*-value
Ecotype	1	0.0071	0	13.58	0	226.25	0	0.00241	0	0.00002	0.216
Salt stress	1	0.0036	0	32.37	0	131.36	0	0.00241	0	0.00108	0
Nitric oxide	1	0.0012	0	1.74	0.0086	8,741.06	0	0.00009	0.0004	0.00884	0
Ecotype*salt	1	0.0006	0.0001	0.34	0.2162	412.87	0	0.00049	0	0.00033	0
Ecotype*nitric oxide	1	0.0004	0.0008	2.83	0.0013	1,236.88	0	0.00004	0.0134	0.00007	0.0179
Salt*nitric oxide	1	0.0028	0	11.33	0	1,751.26	0	0.00312	0	0.00143	0
Ecotype*salt*nitric oxide	1	0.0003	0.0039	0.37	0.2002	2.69	0.3758	0.00009	0.0004	0.00067	0
Error	24	0.9569		0.46		1.82		0.002385		0.00334	
		MDA	*p*-value	Phenolics	*p*-value	Total soluble proteins	*p*-value	CAT	*p*-value	POD	*p*-value
Ecotype	1	0.0601400	0	0.00003	0.2497	0.002	0	1.00E−07	0.9694	0.0921	0
Salt stress	1	0.0025000	0	0.03432	0	0.009	0	4.30E−03	0	0.0653	0
Nitric oxide	1	0.0052400	0	0.01095	0	0.001	0.1534	4.01E−02	0	0.0822	0
Ecotype*salt	1	0.0000300	0.0526	0.00551	0	0.002	0	1.65E−02	0	0.0004	0.3486
Ecotype*nitric oxide	1	0.0000400	0.0308	0.00451	0	0.000	0.3474	1.41E−02	0	0.0002	0.4601
Salt*nitric oxide	1	0.0000002	0.873	0.00005	0.1534	0.015	0	1.45E−01	0	0.2012	0
Ecotype*salt*nitric oxide	1	0.0001100	0.001	0.01428	0	0.002	0	3.73E−04	0.0541	0.0049	0.0025
Error	24	0.0028600		0.004796		0.008		9.54E−03		0.0206	
		APX	*p*-value	Na^+^	*p*-value	K^+^	*p*-value	Ca^2+^	*p*-value	Cl^−^	*p*-value
Ecotype	1	0.002	0.396	1.48	0.32	41,018.92	0.07	0.20	0.12	8.15	0.85
Salt stress	1	0.005	0.141	7.16	0.04	6,356.56	0.46	0.01	0.79	0.29	0.97
Nitric oxide	1	0.034	0.001	0.36	0.62	703.22	0.81	0.01	0.70	131.81	0.44
Ecotype*salt	1	0.003	0.231	0.26	0.68	9.19	0.98	0.00	0.94	331.19	0.22
Ecotype*nitric oxide	1	0.000	0.735	0.01	0.93	2,547.73	0.64	0.02	0.58	4.86	0.88
Salt*nitric oxide	1	0.135	0.000	0.02	0.90	1,879.00	0.69	0.12	0.22	35.82	0.69
Ecotype*salt*nitric oxide	1	0.019	0.007	0.01	0.95	625.43	0.82	0.50	0.02	11.35	0.82
Error	24	0.047		1.44		11,374.30		0.08		212.96	

RMP, relative membrane permeability; MDA, malondialdehyde; CAT, catalase; POD, peroxidase; APX, ascorbate peroxidase.

**Figure 2:**
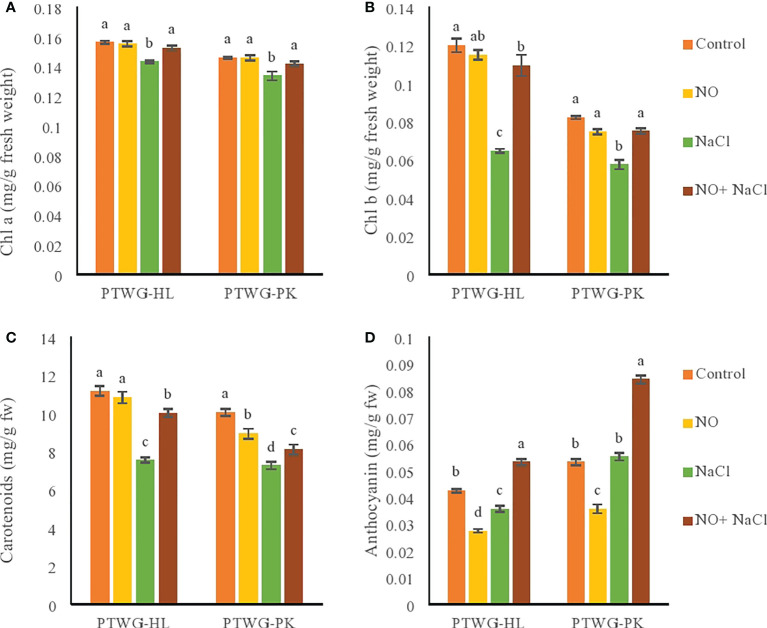
Shows chlorophyll a, chlorophyll b, carotenoids and anthocyanin contents of forty-two days old ecotypes of turnip (*Brassica rapa* L.) under sodium chloride and nitric oxide stress. **(A)** Chlorophyll a, **(B)** Chlorophyll b, **(C)** Carotenoids, **(D)** Anthocyanins. Means with the same letters on each bar do not differ significantly at the 5% level.

In vegetable crops such as turnip, salt stress causes degradation of the chlorophyll and carotenoid contents (Colla et al., 2013a). The contents of chlorophyll a and b were decreased as an effect of salt stress in ecotypes PTWG-HL and PTWG-PK (**Figure 2**). Similar results were also reported in cotton (Dong et al., 2014), rice cultivar (Habib et al., 2013), and *Catharanthus roseus* (Jaleel et al., 2008). The decreased content of chlorophyll under salt stress is related to an increase in the content of Na^+^, which is harmful for many biomolecules (Ashraf et al., 2010a). The use of NO can limit the leakage of ions and loss of chlorophyll. NO application increased the chlorophyll a and b contents in ecotypes PTWG-HL and PTWG-PK. These findings have also been previously reported in cotton (Liu et al., 2014), tomato (Wu et al., 2010), and soybean (Noriega et al., 2007). The application of NO to combat salt stress increased the chlorophyll a and b contents of ecotype PTWG-HL. These results are in line with earlier reports on various plants (Fan et al., 2007; Khan et al., 2012).

The carotenoid gene expression pathway is affected by different abiotic stresses, such as water, light, high temperature, cold, and salinity (Moshtaghi et al., 2011). Similarly, in the present study, salt stress decreased the carotenoid content in ecotype PTWG-HL. Similar results were found in maize (Cha-Um and Kirdmanee, 2009). Under salt stress, the carotenoid content increased in ecotype PTWG-PK. Similar observations were reported in lettuce (Kim et al., 2008) and buckwheat (Lim et al., 2012). Treatment with NO in ecotype PTWG-HL elevated the carotenoid content of plants, as found earlier in chickpeas (Ahmad et al., 2016). Carotenoids are associated with photosynthesis as a factor of the light harvesting system, while salinity reduces photosynthesis (Chartzoulakis and Klapaki, 2000). The combination of salt stress and NO treatment increased the carotenoid content in ecotype PTWG-HL. The same results have been found in tomato (Ali and Ismail, 2014), wheat (Singh et al., 2008), and in *Glycine max* L. (Simaei et al., 2011). However, in this study, ecotype PTWG-PK exhibited a contradictory response.

Salt stress affects the anthocyanin concentration in plants differently. Ecotype PTWG-HL showed a decreased anthocyanin content under salinity stress compared to control plants, similar to that in the tomato cultivar Aft (Anthocyanin fruit), while ecotype PTWG-PK showed increased anthocyanins under salinity stress. Similar results were found in the fruits of the tomato cultivar SB (Sun Black) (Borghesi et al., 2011). The production of anthocyanins in plants could be the reason for their resistance to environmental stresses. Treatment with NO decreased the anthocyanin content of ecotype PTWG-HL. Similar results were also reported in Chinese winter jujube treated with NO (Zhu et al., 2009). On the contrary, ecotype PTWG-PK showed an increased anthocyanin content, similar to that in blackberries (Perkins Veazie and Collins, 2002) and strawberries (Zheng et al., 2007). The use of NO can affect the biosynthetic pathways of anthocyanin and can act against salt stress (Chae et al., 2003). Treatment of salt stress with NO increased the anthocyanin content of PTWG-HL compared to control plants. This was also observed in soybean plants (Simaei et al., 2012).

Cell membrane damage is one of the early effects exhibited by plants under salt stress (Liu et al., 2005). In this study, under salt stress, the RMP increased in ecotypes PTWG-HL and PTWG-PK compared to control plants. The same was found in cucumber (Fan et al., 2007) and maize (Hichem and Mounir, 2009). NO application decreased the membrane permeability in both ecotypes in this study, which has also been described previously in chickpeas (Sheokand et al., 2008).

**Figure 3:**
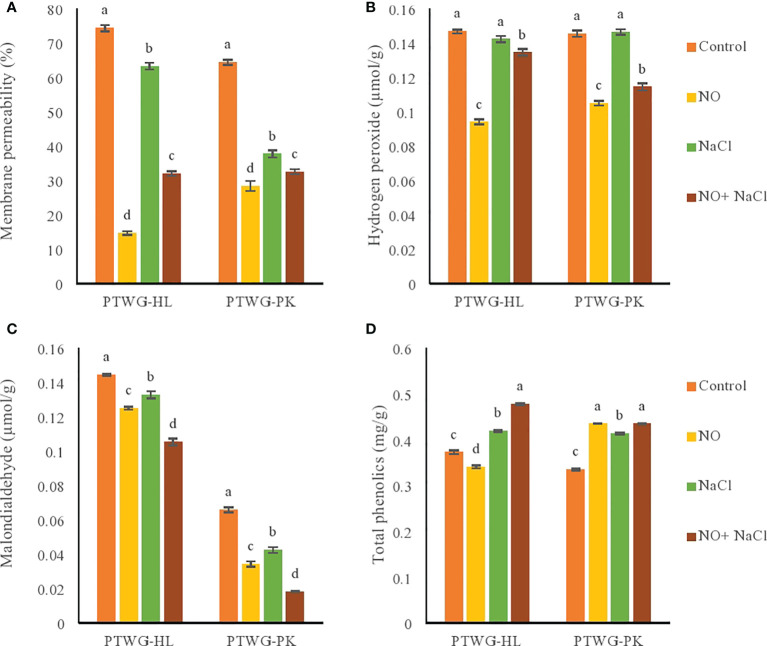
Shows membrane permeability, hydrogen peroxide, malondialdehyde and total phenolic content of forty-two days old ecotypes of turnip (*Brassica rapa* L.) under sodium chloride and nitric oxide stress. **(A)** Membrane permeability, **(B)** Hydrogen peroxide, **(C)** Malondialdehyde, **(D)** Total phenolics. Means with the same letters on each bar do not differ significantly at the 5% level.

Salt stress increased the MDA content in ecotypes PTWG-HL and PTWG-PK (**Figure 3**), as also reported in maize (Gunes et al., 2007) and green beans (Yasar et al., 2008), which suggests damage in plant cells. Under NO treatment, the MDA content in both ecotypes decreased. A similar response was shown in rice (Hung and Kao, 2003). The combination of NO treatment and salt stress also decreased the MDA content in both ecotypes. Similar effects were found in cucumber (Fan et al., 2007) and barley (Beligni et al., 2002). The reduction in MDA content can be due to the low levels of H_2_O_2_. The role of H_2_O_2_ is crucial in the antioxidant response system and in physiological processes (Mittler, 2002). A lot of studies have suggested the increased concentration of H2O2 under abiotic stress (Neill et al., 2002a; Kacperska, 2004). The production rate of H_2_O_2_ is dependent on the duration and level of stress (Slesak et al., 2003). In the present study, the application of salt stress increased the H_2_O_2_ concentration in ecotypes PTWG-HL and PTWG-PK compared to control plants. This has likewise been reported in wheat (Sairam et al., 2005). Previous studies revealed that a high concentration of salt in soil increases the reactive oxygen species (ROS) in plants, which damaged important macromolecules including DNA, the production of H_2_O_2_, and the generation of lipid hydroperoxides, causing changes in the membrane. In this study, NO treatment decreased the concentration of H_2_O_2_ in both ecotypes. This was also previously found in maize (Sun et al., 2007) and wheat (Tian and Lei, 2006). The combination of salt stress and NO treatment also decreased the H_2_O_2_ in both ecotypes. This result has been reported in wheat (Hasanuzzaman et al., 2011) and strawberry (Kaya et al., 2019).

Salt stress affects the phenylpropanoid pathway *via* the assembly of endogenous jasmonic acid (Pedranzani et al., 2007). However, the buildup of phenolic compounds in plants due to salt stress depends on the plant species. In the present study, the total phenolic content increased in ecotype PTWG-HL under salt stress. The same findings were reported in buckwheat (Lim et al., 2012) and maize (Hichem and Mounir, 2009). However, ecotype PTWG-PK showed lower phenolic content compared to control plants (**Figure 3**). The same findings were noted in lettuce (Kim et al., 2008) and sorghum (Kafi et al., 2011). Treatment with NO decreased the phenolic compounds in ecotypes PTWG-HL and PTWG-PK, with the same results also found in longan fruit (Duan et al., 2007). Salt stress and NO treatment increased the phenolic compounds in both ecotypes. Similar results were also found in *Crocus sativus* (Babaei et al., 2019).

**Figure 4:**
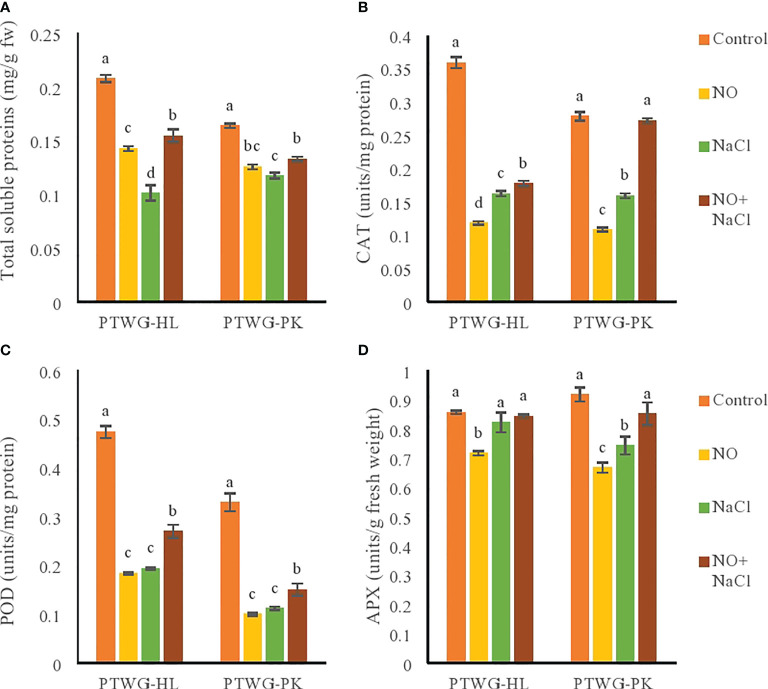
Shows total soluble proteins, catalase, peroxidase and ascorbate peroxidase activity of fortytwo days old ecotypes of turnip (*Brassica rapa* L.) under sodium chloride and nitric oxide stress. **(A)** Total soluble proteins, **(B)** CAT, **(C)** POD, **(D)** APX. Means with the same letters on each bar do not differ significantly at the 5% level.

The mechanism of protein synthesis is affected by salt stress. Total soluble proteins are an indicator of the physiological status of plants (Chen et al., 2009). In the present study, salinity decreased the total soluble proteins in ecotype PTWG-HL (**Figure 4**), with similar results reported in tomato cultivars (Doganlar et al., 2010) and in bean species (Yurekli et al., 2004). The application of NO increased the total soluble proteins in ecotypes PTWG-HL and PTWG-PK compared to plants subjected to salt stress (**Figure 4**). This has been previously observed in *Arachis hypogaea* L. (Kobeasy et al., 2011) and spinach (Jin et al., 2009). Salt stress along with NO application exhibited increased total soluble proteins in both ecotypes. The same results were found in chickpea (Ahmad et al., 2016).

The antioxidant enzymes CAT, POD, and superoxide dismutase (SOD) are ROS scavengers (**Figure 4**). Under salt stress, the concentrations of ROS exceed their threshold levels and are scavenged by antioxidant enzymes (Wang et al., 2013). Salt stress decreased the CAT enzyme activity in ecotype PTWG-HL, which has been reported in cotton seedlings (Liu et al., 2014), *Oryza sativa* L. seedlings (Mishra et al., 2013), and *Phaseolus vulgaris* (Jungklang et al., 2004). The present study showed that, under salt stress, the activities of POD and APX decreased, which was also found previously in tomato (Manai et al., 2014) and sugarcane (Patade et al., 2012). NO application decreased the POD activity in ecotypes PTWG-HL and PTWG-PK compared to control plants. Similar results have been found in cut lettuce (Wills et al., 2008) and in peeled bamboo shoots (Yang et al., 2010). The protective role of NO is primarily based on its ability to maintain the redox homeostasis in plant cells and regulate the toxicity caused by ROS under salt stress. The combined application of NO and salt stress increased the CAT activity in ecotype PTWG-HL. This result has also been reported in sunflower plants (Ramadan et al., 2019) and wheat seedlings (Ruan et al., 2004; Zheng et al, 2009). CAT and POD act as SOD in limiting the negative effects of salt stress in plants. Increased POD and APX activities were observed in ecotype PTWG-HL. Similar results were also found in *Solanum lycopersicum* (Manai et al., 2014), soybean (Simaei et al., 2011), and chickpea (Sheokand et al., 2010).

The concentrations of different ions provide a basis for the physiological response of plants in relation to their development (Wang et al., 2003). Increased uptake of salt causes the production of specific ionic toxicants, such as high concentrations of Na^+^ and Cl^−^, which decrease the uptake of necessary nutrients including nitrogen, calcium, phosphorus, and potassium (Zhu, 2002). Saline soils and saline waters contain abundant amounts of Na^+^ and Cl^−^ ions, which cause ionic toxicity in plants, in turn causing a reduction in the yield potential (Munns and Tester, 2008).

The application of salt stress increased the sodium content in ecotypes PTWG-HL and PTWG-PK compared to control plants. Similar results were reported in cotton seedlings (Dong et al., 2014) and in *Gerbera jamesonii* L. (Don et al., 2010). However, NO treatment of both ecotypes decreased the sodium content compared to control plants. Similarly, a reduction in Na content was found in *Triticum aestivum* L. (Kausar et al., 2013) and in maize (Zhang et al., 2004). The application of NO and salt stress also caused a decrease in the sodium content of both ecotypes. Comparable findings were reported in *Zea Z. mays* L. (Kaya et al., 2015) and *O. sativa* L. (Habib and Ashraf, 2014).

**Figure 5:**
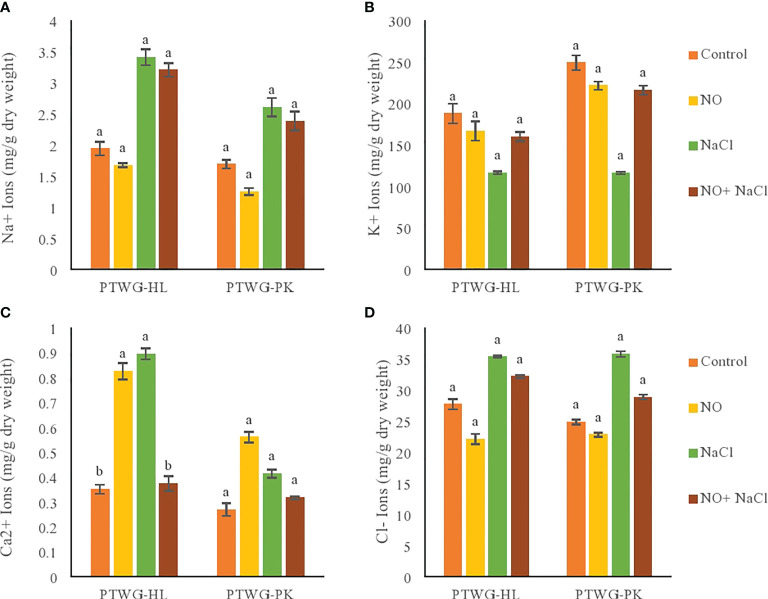
Shows ionic content of forty-two days old ecotypes of turnip (*Brassica rapa* L.) under sodium chloride and nitric oxide stress. **(A)** Na^+^, **(B)** K^+^, **(C)** Ca^2+^, **(D)** Cl^-^. Means with the same letters on each bar do not differ significantly at the 5% level.

Calcium and potassium ions are crucial for the balanced growth of plants, but under salt stress, their levels are markedly reduced due to the accumulation of the toxic ions Na^+^ and Cl^−^ (Acosta-Motos et al., 2017). Salt stress reduced the potassium content in ecotype PTWG-HL compared to control plants (**Figure 5**). Similar results were found in *Matricaria recutita* L. (Baghalian et al., 2008). The application of NO also increased the potassium content in ecotypes PTWG-HL and PTWG-PK. Similar studies have been conducted in maize plants (Kaya et al., 2019) and perennial ryegrass (Wang et al., 2013). The high concentration of K^+^ may be due to the enhanced transcription of K^+^ channels upon NO treatment (Xia et al., 2014). The application of NO under salt stress increased the potassium content in ecotype PTWG-HL compared to control plants, which was also previously observed in strawberry plants (Kaya et al., 2019).

Salt stress decreased the calcium content in ecotype PTWG-PK (**Figure 5**), also previously described in soybean plants (Amirjani, 2010) and *Pistacia vera* (Rahneshan et al., 2018). NO treatment increased the concentration calcium in ecotype PTWG-HL compared to control plants. Similar findings have been reported in perennial ryegrass (Wang et al., 2013) and cotton seedlings (Liu et al., 2014). Treatment with NO under salt stress increased the calcium content in ecotype PTWG-PK, with similar findings reported in cotton seedling (Liu et al., 2013) and *Linum usitatissimum* (Khan et al., 2010).

Salt stress treatment increased the concentration of Cl^−^ in ecotype PTWG-HL compared to control plants (**Figure 5**), with the same results also noted in *Atriplex griffithii* (Parihar et al., 2015) and maize (Turan et al., 2010). Cl^−^ toxicity in soil is one of the causes of growth retardation in plants, which occurs due to high salinity (Tavakkoli et al., 2011). The treatment of plants with NO decreased the Cl^−^ content in ecotype PTWG-HL compared to control plants, with similar results reported in *T. aestivum* (Kausar et al., 2013). The combination of salt stress and NO treatment decreased the chloride content in ecotype PTWG-PK compared to control plants, also reported previously in cotton seedlings (Liu et al., 2014).

## Conclusion

Salt toxicity reduced the growth of the *B. rapa* ecotypes in this study. However, the application of NO reduced the toxic effect of salt stress by improving the activity of the antioxidative defense system. NO treatment also reduced the MDA and 24H_2_O_2_ contents in *B. rapa* seedlings. Conclusively, salt stress alleviation in *B. rapa* was ascribed to the enhanced activity of antioxidant enzymes, in addition to the increment in ionic contents. The *B. rapa* ecotype PTWG-PK showed better growth with supplementation of NO compared to the PTWG-HL ecotype. Therefore, it is recommended that the molecular mechanisms associated with NO-induced tolerance in various crops be exploited.

## Data availability statement

The original contributions presented in the study are included in the article/supplementary material. Further inquiries can be directed to the corresponding authors.

## Author contributions

AB conducted the research and wrote the original draft. ZN supervised the whole research. MR and MA interpreted the data for formal analysis. FZ, AAS and ANS reviewed the original draft. MJ and NA revised the manuscript. NRA and MEH reviewed the final manuscript. All authors contributed to the article and approved the submitted version.

## Acknowledgments

We would like to acknowledge Alexandria University, Alexandria, Egypt, and King Abdullah University of Science and Technology, Saudi Arabia, for supporting the research.

## Conflict of interest

The authors declare that the research was conducted in the absence of any commercial or financial relationships that could be construed as a potential conflict of interest.

## Publisher’s note

All claims expressed in this article are solely those of the authors and do not necessarily represent those of their affiliated organizations, or those of the publisher, the editors and the reviewers. Any product that may be evaluated in this article, or claim that may be made by its manufacturer, is not guaranteed or endorsed by the publisher.
